# Glucokinase Regulatory Protein Gene Polymorphism Affects Liver Fibrosis in Non-Alcoholic Fatty Liver Disease

**DOI:** 10.1371/journal.pone.0087523

**Published:** 2014-02-03

**Authors:** Salvatore Petta, Luca Miele, Elisabetta Bugianesi, Calogero Cammà, Chiara Rosso, Stefania Boccia, Daniela Cabibi, Vito Di Marco, Stefania Grimaudo, Antonio Grieco, Rosaria Maria Pipitone, Giulio Marchesini, Antonio Craxì

**Affiliations:** 1 Sezione di Gastroenterologia, Dipartimento Biomedico di Medicina Interna e Specialistica, University of Palermo, Palermo, Italy; 2 Institute of Internal Medicine, School of Medicine, Catholic University of the Sacred Heart, Rome, Italy; 3 Division of Gastroenterology, Dept. of Medical Sciences, University of Turin, Turin, Italy; 4 Cattedra di Anatomia Patologica, University of Palermo, Palermo, Italy; 5 Dipartimento di Medicina e Gastroenterologia, “Alma Mater Studiorum,” Università di Bologna, Bologna, Italy; University of Modena & Reggio Emilia, Italy

## Abstract

**Background and Aims:**

Variant in glucokinase regulatory protein (GCKR), associated with lipid and glucose traits, has been suggested to affect fatty liver infiltration. We aimed to assess whether GCKR rs780094 C→T SNP influences the expression of steatosis, lobular inflammation and fibrosis in NAFLD patients, after correction for PNPLA3 genotype.

**Methods:**

In 366 consecutive NAFLD patients (197 from Sicily, and 169 from center/northern Italy), we assessed anthropometric, biochemical and metabolic features; liver biopsy was scored according to Kleiner. PNPLA3 rs738409 C>G and GCKR rs780094 C>T single nucleotide polymorphisms were also assessed.

**Results:**

At multivariate logistic regression analysis in the entire NAFLD cohort, the presence of significant liver fibrosis (>F1) was independently linked to high HOMA (OR 1.12, 95% CI 1.01–1.23, *p* = 0.02), NAFLD activity score ≥5 (OR 4.09, 95% CI 2.45–6.81, *p*<0.001), and GCKR C>T SNP (OR 2.06, 95% CI 1.43–2.98, *p*<0.001). Similar results were observed considering separately the two different NAFLD cohorts. GCKR C>T SNP was also associated with higher serum triglycerides (ANOVA, p = 0.02) in the entire cohort.

**Conclusions:**

In patients with NAFLD, GCKR rs780094 C>T is associated with the severity of liver fibrosis and with higher serum triglyceride levels.

## Introduction

Non-alcoholic fatty liver disease (NAFLD) is a frequent and growing cause of chronic liver disease [1], [2], affecting about 20%–30% of the general population worldwide [3]. Patients with NAFLD, and especially those with non-alcoholic steatohepatitis (NASH), are at risk of progression to cirrhosis and its complications [1], [4], presenting also a high rate of cancer and cardiovascular events [5] compared to subjects without fatty liver. Classical risk factors for liver disease severity and its progression are obesity, insulin resistance (IR) and necroinflammation [6]–[9].

The above-mentioned conventional risk factors do not entirely explain the occurrence and severity of NAFLD, suggesting that a genetic background might also modulate the spectrum of liver disease and its progression. Accordingly, the severity of disease has been variably associated with different single nucleotide polymorphisms (SNPs) in genes involved in metabolic homeostasis, inflammation, oxidative stress and fibrogenesis [10]. In this context, the patatin-like phospholipase-3 (*PNPLA3)/adiponutrin*, rs738409 C>G SNP remains the most validated risk gene [11]–[18].

Besides the classical PNPLA3, a recent genome wide study identified other genetic variants associated with computerized tomography (TC)-proven hepatic steatosis in individuals of European ancestry, and validated the results in 592 subjects with biopsy-proven NAFLD from the NASH Clinical Research Network (NASH CRN) database [19]. Among these gene variants, the glucokinase regulatory protein (GCKR) has been further confirmed as linked with steatosis (identified either by ultrasonography, by magnetic resonance, or by computed tomography) in children, in obese patients, and in populations of different ethnicity [20]–[22]. GCKR seems to interfere with glucose and lipid homeostasis by regulating glucose storage/disposal and by providing substrates for de novo lipogenesis via inhibition of glucokinase, but its potential association with the severity of liver damage has never been tested

Having this in mind, the main outcome of this study was to assess whether GCKR rs780094 was associated with the histological features of liver damage in patients with biopsy-proven NAFLD, after correction for PNPLA3 genotype.

## Patients and Methods

### Patients

We analyzed data from 366 prospectively recruited Italian patients with a clinical and histological diagnosis of NAFLD, and with blood samples available for genetic analyses. The study cohort included 197 patients from the Gastrointestinal & Liver Unit of the Palermo University Hospital, and 169 central/northern Italy patients from the Department of Internal Medicine Catholic University of the Sacred Heart Rome (n = 114), and from the Gastro-hepatology Division of the University Hospital Torino (n = 55). Other causes of liver disease were ruled out, including alcohol intake (>20 g/day) evaluated by a questionnaire, viral and autoimmune hepatitis, hereditary hemochromatosis and alpha1-antitrypsin deficiency. Patients with advanced cirrhosis, hepatocellular carcinoma and current use of steatosis inducing drugs were excluded.

The study was carried out in accordance with the principles of the Helsinki Declaration, and with local and national laws. Approval was obtained from the hospital Internal Review Boards and their Ethics Committees (UOC Gastroenterologia, AOUP Policlinico of Palermo, Institute of Internal Medicine, Catholic University of the Sacred Heart, Rome, and Division of Gastro-Hepatology, San Giovanni Battista Hospital, University of Torino), and written informed consent for the study was obtained from all patients.

### Clinical and Laboratory Assessment

Clinical and anthropometric data were collected at the time of liver biopsy. Body mass index (BMI) was calculated on the basis of weight in kilograms and height in meters. The diagnosis of arterial hypertension was based on the following criteria: systolic blood pressure ≥135 mm Hg and/or diastolic blood pressure ≥85 mm Hg (measured three times within 30 minutes, in the sitting position and using a brachial sphygmomanometer), or use of blood-pressure-lowering agents. The diagnosis of type 2 diabetes was based on the revised criteria of the American Diabetes Association, using a value of fasting cut-off value of blood glucose ≥126 mg/dl on at least two occasions [23]. In patients with a previous diagnosis of type 2 diabetes, current therapy with insulin or oral hypoglycemic agents was documented.

A 12-hour overnight fasting blood sample was drawn at the time of biopsy to determine serum levels of ALT, total cholesterol, HDL-cholesterol, triglycerides, plasma glucose and insulin concentrations. IR was assessed by homeostasis model assessment (HOMA), using the following equation [24]: Insulin resistance (HOMA-IR) = Fasting insulin (µU/mL)×Fasting glucose (mmol/L)/22.5. [25]. HOMAbeta was also assessed as expression of pancreatic beta-cell function [26]

### Genetic Analyses

DNA was purified using the QIAmp blood Mini Kit (Qiagen, Mainz, Germany) and DNA samples were quantified using spectrophotometric determination. Genotyping for PNPLA3 (*rs738409)*, *and GCKR (rs780094)* was carried out using the TaqMan SNP genotyping allelic discrimination method (Applied Biosystems, Foster City, CA, USA). Commercial genotyping assays were available for the following SNPs: *rs738409* (cat. C_7241_10), rs780094 (cat. C_2862873_10).

The genotyping call was done with SDS software v.1.3.0 (ABI Prism 7500, Foster City, CA, USA). Genotyping was conducted in a blinded fashion relative to patient characteristics.

### Assessment of Histology

Slides were coded and read at each clinical center by one expert pathologist, who was unaware of patients' identity and history. A minimum 15 mm-length of the biopsy specimen or the presence of at least 10 complete portal tracts was required [27]. Steatosis was assessed as the percentage of hepatocytes containing fat droplets (minimum 5%) and evaluated as a continuous variable. Kleiner classification [28] was used to compute steatosis, balloning and lobular inflammation, and to stage fibrosis from 0 to 4. NASH was considered to be present when the NAFLD activity score (NAS) was ≥5 [28].

### Statistics

Continuous variables were summarized as mean ± standard deviation, and categorical variables as frequency and percentage. The t-test, ANOVA test, and chi-square test were used when appropriate.

Multiple logistic regression models were used to assess the factors independently associated with severe steatosis, NAS ≥5, and significant fibrosis. In the first model, the dependent variable was steatosis, coded as 0 = mild-moderate (steatosis grade 1–2), 1 = severe (steatosis grade 3). In the second model, the dependent variable was NAS ≥5, coded as 0 = NAS<5 or 1 = NAS ≥5. In the third model, the dependent variable was fibrosis, coded as 0 = no significant fibrosis (F0–F1) or 1 = significant fibrosis (>F1).

As candidate risk factors, we selected age, gender, BMI, the baseline levels of ALT, triglycerides, total and HDL cholesterol, blood glucose, insulin, HOMA score, the presence of diabetes, arterial hypertension, PNPLA3 rs738409, GCKR rs780094, steatosis, lobular inflammation and fibrosis.

In all models, in agreement with literature data, we compared patients homozygous for PNPLA3 G risk allele to all other variants [14], while an additive model was used GCKR C>T SNP [19].

To avoid the effect of colinearity, diabetes, HOMA score, blood glucose and insulin levels, or ALT levels and lobular inflammation were not included in the same multivariate model. Variables associated with the dependent variable at univariate analysis (probability threshold, p≤0.10) were included in the multivariate regression models; PNPLA3 and GCKR SNPs, were forced into the models when not significant associated to the tested dependent variable. Regression analyses were performed using PROC LOGISTIC, PROC REG, and subroutines in SAS [29].

## Results

### Patient Features and Histology

The baseline characteristics of the 197 Sicilian and the 169 Center/Northern Italian NAFLD patients are shown in Table 1. Patients from Sicily were slightly older and with a moderately lower prevalence of males, were more likely to be obese and to have more severe steatosis and lobular inflammation compared to Center/Northern Italian patients. Nevertheless, histological staging was similar in the two cohorts and significant fibrosis (>F1) was present in approximately half of the patients.

**Table 1:** Baseline Demographic, Laboratory, Metabolic, and Histological Features of 366 Italian Patients with Non-alcoholic Fatty Liver Disease.

Variable	Non-alcoholic Fatty Liver Disease (Sicily n = 197)	Non-alcoholic Fatty Liver Disease (Center/Northern Italy n = 169)	P value
**Mean Age** – **years**	45.0±13.3	42.2±11.2	0.03
**Male Gender**	131 (66.5)	130 (76.9)	0.03
**Mean Body Mass Index** – **kg/m^2^**	29.7±4.6	27.8±4.1	<0.001
**Alanine Aminotransferase – IU/L**	79.4±55.1	77.8±53.3	0.78
**Arterial Hypertension**	46 (23.3)	47 (27.8)	0.21
**Type 2 Diabetes**	28 (14.2)	22 (11.2)	0.39
**Cholesterol – mg/dL**	205.8±44.8	203.4±45.2	0.74
**HDL Cholesterol – mg/dL**	49.0±15.4	47.7±11.3	0.38
**Triglycerides – mg/dL**	147.6±78.7	142.7±86.9	0.57
**Blood Glucose – mg/dL**	96.1±25.2	94.7±21.5	0.56
**Insulin – µU/mL**	16.5±9.6	14.5±10.6	0.08
**HOMA Score**	4.03±2.96	3.57±3.41	0.18
**PNPLA3 rs738409 polymorphism**			
C/C	62 (31.5)	54 (31.9)	
C/G	94 (47.7)	79 (46.7)	
G/G	41 (20.8)	36 (21.4)	0.98
**GCKR rs780094 polymorphism**			
C/C	32 (16.2)	28 (16.6)	
C/T	99 (50.3)	80 (47.3)	
T/T	66 (33.5)	61 (36.1)	0.98
**Histology**			
**Lobular inflammation**			
2–3			
**Balloning**	83 (42.1)	20 (11.8)	<0.001
1–2	181 (91.8)	122 (71.7)	<0.001
**Steatosis grade**			
1 (5%–33%)	72 (36.6)	76 (44.9)	
2 (>33%–66%)	62 (31.5)	62 (36.7)	
3 (>66%)	63 (31.9)	31 (18.4)	0.01
**NAS≥5**	116 (58.8)	41 (24.2)	<0.001
**Stage of Fibrosis**			
2–4	86 (43.6)	72 (42.6)	0.84

Abbreviation: IU, international units; HOMA, homeostasis model assessment; HDL, high density lipoprotein. Data are given as mean ± standard deviation, or as number of cases (%).

The prevalence of GCKR rs780094 CC, CT and TT genotypes was 16.4%, 48.9% and 34.7% in the entire cohort. When split according to center (Sicily vs. Center/Northern Italy) the prevalence of the single SNPs was similar (16.2%, 50.3% and 33.5% in the Sicilian cohort, vs. 16.6%, 47.3% and 36.1%; p = 0.98). Similarly, the prevalence of PNPLA3 rs738409 CC, CG and GG genotypes was 31.5%, 47.7% and 20.8% in the Sicilian cohort, and 31.9%, 46.7% and 21.4% in the Center/Northern Italy cohort (p = 0.98).

Genetic frequencies of the two polymorphisms fit with Hardy–Weinberg equilibrium.

### Influence of GCKR Genotype on Metabolic and Biochemical Parameters

The association of the rs780094 GCKR C→T genotype with anthropometric, metabolic and biochemical parameters in the entire cohort is shown in Table 2. The only significant association was between the TT genotype and higher triglyceride levels, although independent of age, gender distribution, and BMI. When split according to center, broadly similar data were observed in the Center/Northern Italian cohort, where an association between GCKR TT genotype and higher blood glucose levels was also observed (85.5±11.6 for CC, 96.7±24.4 for CT, 96.4±20 for TT; p = 0.03). By contrast, in the Sicilian cohort the rs780094 genotype was not associated with abnormal triglyceride levels.

**Table 2:** Association of the rs780094 C→T GCKR SNP with anthropometric, biochemical, metabolic and histological features in 366 Patients with Non-alcoholic Fatty Liver Disease.

Variable	GCKR CC N = 60	GCKR CT n = 179	GCKR TT n = 127	Univariate Analysis *p* value°
**Mean age** – **years**	42.2±13.0	43.3±12.6	44.9±11.8	0.33
**Males**	48	127	86	0.22
**Mean body mass index** – **kg/m^2^**	28.5±4.6	28.7±4.4	29.1±4.5	0.61
**Alanine aminotransferase – IU/L**	81.6±70.6	81.4±52.1	73.4±48.0	0.40
**Cholesterol – mg/dL**	198.6±47.9	202.4±40.9	209.5±48.5	0.23
**HDL cholesterol – mg/dL**	50.2±18.2	48.9±13.2	47.1±12.2	0.32
**Triglycerides – mg/dL**	139.8±83.3	138.0±69.8	158.3±96.3*	0.09
**Blood glucose – mg/dL**	91.4±18.6	96.6±27.7	95.8±18.5	0.32
**Insulin – µU/mL**	15.1±9.8	16.0±9.1	15.4±11.5	0.78
**HOMA-score**	3.65±3.23	3.96±3.03	3.70±3.05	0.68
**HOMAbeta %**	144.4±63.7	144.6±71.5	131.8±61.0	0.25
**Diabetes**	8	21	18	0.81
**Arterial hypertension**	14	41	38	0.35
**Steatosis grade 3**	13	44	37	0.49
**Lobular Inflammation Grade 2–3**	17	53	34	0.86
**Ballooning**	46	151	105	0.39
**NAS ≥5**	24	82	51	0.54
**Fibrosis >F1**	17	71	70	0.001

Data are given as mean ± SD or as number of cases. HDL: high-density lipoprotein; HOMA: homeostasis model assessment.

° by ANOVA;

* p = 0.02 versus GCKR CC/CT.

### Factors Associated with Histological Features

In the entire cohort, multivariate logistic regression analysis showed that high BMI (OR 1.10, 95% CI 1.04–1.117, *p* = 0.001), high HOMA considered as continuous variable (OR 1.08, 95% CI 1.00–1.17, *p* = 0.04), and PNPLA3 GG (OR 1.97, 95% CI 1.11–3.51, *p* = 0.02) were independently associated with severe steatosis (table 3 upper panel). Similar results were observed when analyses were split by center (table 3 upper panel).

**Table 3:** Association of the GCKR rs780094 genotype and liver damage as evaluated by unadjusted and adjusted models in 366 Patients with Non-alcoholic Fatty Liver Disease.

	Sicilian Cohort n = 197		Center/northern Italy Cohort n = 169		Combined n = 366	
Variable	OR (95% C.I.)	P value	OR (95% C.I.)	P value	OR (95% C.I.)	P value
	**Steatosis (1 vs 2 vs 3)**					
	**Unadjusted Model**	**Adjusted Model**	**Unadjusted Model**	**Adjusted Model**	**Unadjusted Model**	**Adjusted Model**
**Females**	-	-	-	-	1.94 (1.18–3.20) 0.009	1.43 (0.83–2.44) 0.19
**Mean BMI** – **kg/m^2^**	1.09 (1.02–1.17) 0.006	1.08 (1.00–1.16) 0.03	1.15 (1.05–1.27) 0.003	1.12 (1.01–1.25) 0.02	1.13 (1.07–1.19) <0.001	1.10 (1.04–1.17) 0.001
**HOMA-score**	1.13 (1.01–1.25) 0.02	1.08 (0.97–1.21) 0.14	1.16 (1.03–1.29) 0.01	1.08 (0.96–1.22) 0.17	1.15 (1.06–1.24) <0.001	1.08 (1.00–1.17) 0.04
**GCKR CC vs CT vs TT**	1.23 (0.79–1.91) 0.35	1.19 (0.75–1.90) 0.44	1.27 (0.72–2.25) 0.40	1.37 (0.74–2.52) 0.31	1.22 (0.87–1.73) 0.24	1.22 (0.85–1.76) 0.27
**PNPLA3 CC vs CG vs GG**	2.19 (1.08–4.45) 0.02	2.12 (1.02–4.41) 0.04	2.48 (1.05–5.83) 0.03	2.50 (1.03–6.05) 0.04	2.24 (1.31–3.83) 0.003	1.97 (1.11–3.51) 0.02
	**NAS≥5**					
**Mean age** – **years**	-	-	-	-	1.01 (1.00–1.03) 0.05	0.99 (0.97–1.00) 0.79
**Females**	2.47 (1.30–4.69) 0.006	1.69 (0.83–3.42) 0.14	3.98 (1.83–8.64) <0.001	3.70 (1.62–8.47) 0.002	3.16 (1.97–5.06) <0.001	2.55 (1.51–4.31) <0.001
**Mean BMI** – **kg/m^2^**	1.10 (1.03–1.18) 0.003	1.05 (0.97–1.13) 0.21	1.15 (1.05–1.25) 0.002	1.13 (1.02–1.25) 0.01	1.14 (1.09–1.21) <0.001	1.11 (1.04–1.17) 0.004
**HOMA-score**	1.38 (1.17–1.64) <0.001	1.28 (1.07–1.53) 0.005	1.14 (1.02–1.27) 0.01	1.03 (0.91–1.17) 0.54	1.24 (1.12–1.36) <0.001	1.13 (1.03–1.25) 0.01
**GCKR CC vs CT vs TT**	1.14 (0.75–1.73) 0.52	1.10 (0.69–1.75) 0.67	0.76 (0.46–1.27) 0.30	0.75 (0.43–1.30) 0.31	0.96 (0.71–1.29) 0.79	0.91 (0.65–1.26) 0.58
**PNPLA3 CC/CG vs GG**	3.62 (1.57–8.35) 0.002	3.06 (1.26–7.41) 0.01	2.49 (1.13–5.51) 0.02	2.31 (1.00–5.31) 0.04	2.54 (1.52–4.27) <0.001	2.15 (1.22–3.77) 0.008
	**Fibrosis (0–1 versus 2–4)**					
**Mean age** – **years**	1.03 (1.01–1.06) 0.001	1.03 (1.00–1.05) 0.02	-	-	1.02 (1.01–1.04) 0.001	1.01 (0.99–1.03) 0.28
**Females**	2.34 (1.28–4.29) 0.006	1.44 (0.68–3.03) 0.33	2.73 (1.30–5.71) 0.007	2.06 (0.86–4.91) 0.10	2.47 (1.55–3.93) <0.001	1.40 (0.80–2.45) 0.23
**Mean BMI** – **kg/m^2^**	1.10 (1.03–1.18) 0.002	1.02 (0.94–1.11) 0.52	1.17(1.07–1.28) <0.001	1.12 (1.01–1.23) 0.02	1.12 (1.07–1.18) <0.001	1.05 (0.98–1.11) 0.10
**HOMA-score**	1.31 (1.14–1.52)<0.001	1.15 (1.00–1.32) 0.04	1.16 (1.03–1.32) 0.01	1.06 (0.91–1.23) 0.43	1.24 (1.12–1.36) <0.001	1.12 (1.01–1.23) 0.02
**Arterial hypertension**	-	-	-	-	1.93 (1.20–3.12) 0.007	1.36 (0.76–2.43) 0.29
**NAS≥5**	7.83 (3.93–15.5) <0.001	7.05 (3.22–15.4) <0.001	5.58 (2.55–12.2) <0.001	4.43 (1.80–10.9) 0.001	5.06 (3.23–7.93) <0.001	4.09 (2.45–6.81) <0.001
**GCKR CC vs CT vs TT**	1.82 (1.18–2.81) 0.006	2.07 (1.22–3.49) 0.006	1.75 (1.10–2.76) 0.01	2.23 (1.31–3.82) 0.003	1.77 (1.30–2.44) <0.001	2.06 (1.43–2.98) <0.001
**PNPLA3 CC/CG vs GG**	1.47 (0.73–2.93) 0.27	0.62 (0.26–1.49) 0.29	1.94 (0.92–4.09) 0.07	1.32 (0.54–3.18) 0.53	1.67 (1.01–2.67) 0.04	1.03 (0.56–1.87) 0.92

Unadjusted and adjusted OR were presented for PNPLA3 and GCKR SNPs, and only for clinical, metabolic and histological variables significant at univariate analysis.

PNPLA3 and GCKR SNPs, when not significant, were forced into the models.

In the whole NAFLD cohort, NAS score ≥5 was independently associated with female gender (OR 2.55, 95% CI 1.51–4.31, *p*<0.001), high BMI (OR 1.11 95% CI 1.04–1.17, *p* = 0.004), HOMA (OR 1.13 95% CI 1.03–1.25, *p* = 0.01), and PNPLA3 GG (OR 2.15, 95% CI 1.22–3.77, *p* = 0.008) at multivariate logistic regression analysis (table 3).

Finally, the presence of significant liver fibrosis (>F1) was independently linked to high HOMA (OR 1.12, 95% CI 1.01–1.23, *p* = 0.02), NAS ≥5 (OR 4.09, 95% CI 2.45–6.81, *p*<0.001), and GCKR C>T SNP (OR 2.06, 95% CI 1.43–2.98, *p*<0.001) in the whole study population (table 3 lower panel). When histological variables were removed from the model, >F1 fibrosis was independently linked to female gender (OR 1.77, 95% CI 1.05–2.99, *p* = 0.03), BMI (OR 1.08, 95% CI 1.02–1.14, *p* = 0.008), HOMA (OR 1.16, 95% CI 1.05–1.28, *p* = 0.004), and GCKR C>T SNP (OR 1.88, 95% CI 1.33–2.66, *p*<0.001).

**Figure 1:**
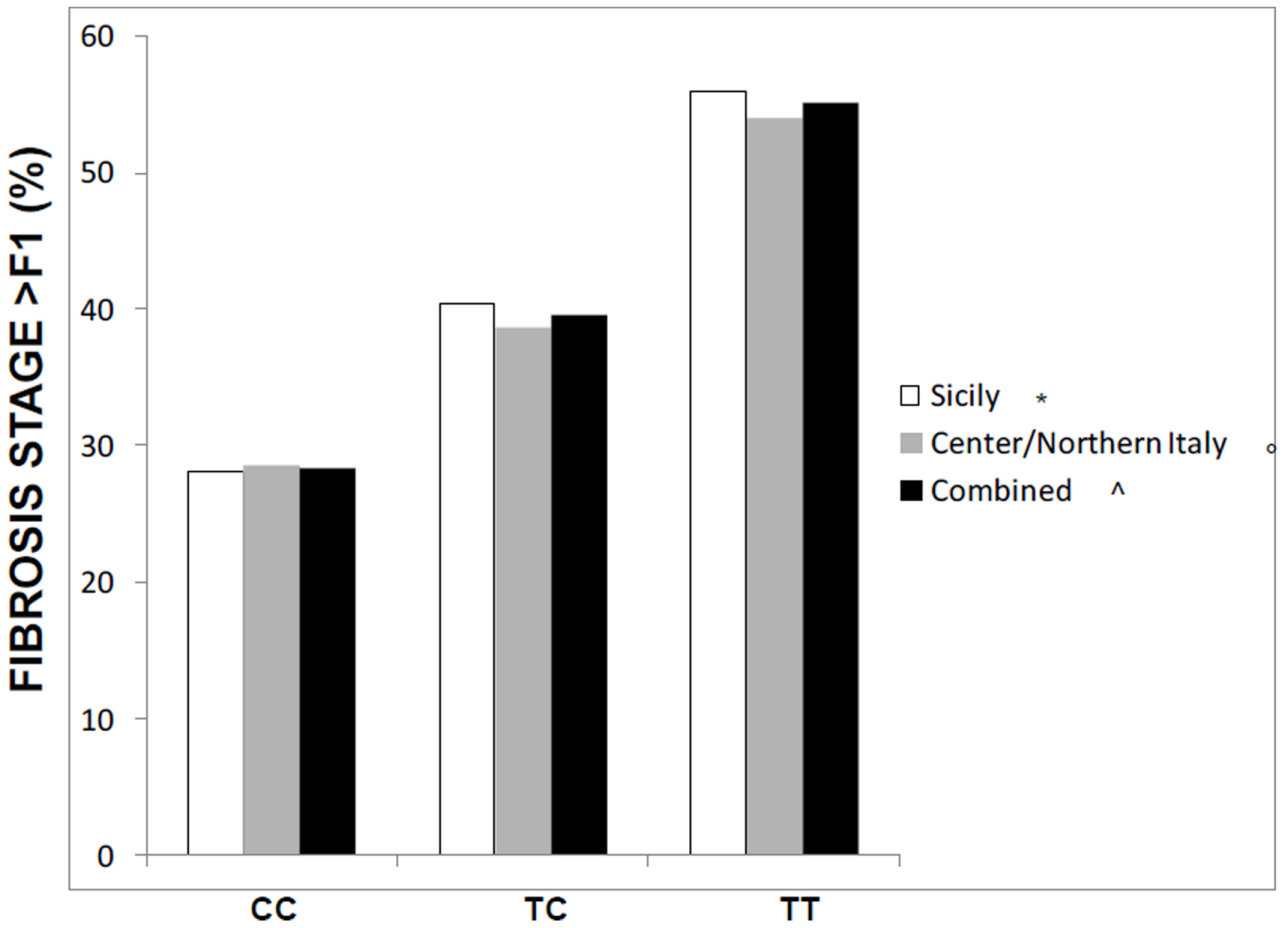
Prevalence of significant fibrosis according to GCKR rs780094 SNP in the Sicilian cohort, in the Center/Northern Italian cohort, and in the combined cohorts of NAFLD patients. *p = 0.02 for prevalence of F2–F4 fibrosis according to GCKR genotype; ° p = 0.04 for prevalence of F2–F4 fibrosis according to GCKR genotype; ∧p = 0.001 for prevalence of F2–F4 fibrosis according to GCKR genotype.

Figure 1 shows the stepwise increased prevalence of significant fibrosis (>F1) according to GCKR genotype. Similar results were observed when the NAFLD cohorts were split by center (table 3 lower panel).

## Discussion

The main finding of this study on a large cohort of Italian patients with NAFLD is the association between GCKR rs780094 C>T SNP and high triglyceride levels and the severity of liver fibrosis, independent of other known risk factors for liver damage

The complex interplay between genetic background and environmental factors in the development of NAFLD [26] is progressively unveiled by the recognition of the role of specific SNPs, such as PNPLA3 C>G SNPs. The GCKR C→T SNPs is also emerging as an important genetic determinant of NAFLD. This trait has been initially associated with imaging-based diagnosis of NAFLD in a population of healthy subjects [19], and the association has been further validated in children and in patients of different ethnicity [20]–[22]. In this study, besides confirming the associations between PNPLA3 C>G SNP and steatosis/lobular inflammation [11]–[15], for the first time we also highlighted the potential impact of GCKR C→T SNP on liver fibrosis in an European cohort of histologically diagnosed NAFLD patients.

Of importance, this association was independent from other well-known determinants and the severity of liver fibrosis showed a stepwise increase from patients carrying one risk allele to those carrying two risk alleles. It is noteworthy that this trend was observed in both tested cohorts. Our findings are in agreement with the evidence of a link between GCKR C→T SNP and higher ALT levels reported by Hernaez et al [22] in the US Health and Nutrition Examination Survey III, and also agree with preliminary unpublished data from FLIP cohort [30], and published data from a small Asiatic population [31] reporting the association between GCKR gene variant and severity of liver damage in NAFLD.

Although this study was not designed to clarify the pathogenic link between GCKR SNPs and liver fibrosis in NAFLD, some hypotheses can be provided. The GCKR gene product, the glucokinase regulatory protein, acts as an inhibitor of glucokinase (GCK) activity, a key liver enzyme for glucose metabolism, whose hepatic concentrations are increased in fatty liver [32], [33]. GCKR rs780094 C>T SNP might facilitate liver fibrogenesis by reducing the inhibitory effect of GCKR, thereby increasing GCK activity. GCKR rs780094 SNP is in linkage disequilibrium with the GCKR rs1260326 SNP, that is associated with an increased GCK liver activity [20], [34]. By inducing *de novo* hepatic lipogenesis and by suppressing hepatic fatty acid oxidation, GCK liver activity prompts the hepatic accumulation of triglycerides, causing organ damage. In addition, GCKR rs780094 SNP might favor liver fibrogenesis along two more pathways: a) by affecting the expression of nearby genes, and specifically by increasing the expression of C2orf16 mRNA in the liver [19]; b) by favoring a low grade chronic inflammation as expressed by higher serum levels of C-reactive protein [35].

In our study we also demonstrated that patient homozygous for the T allele of GCKR had higher serum triglycerides levels, in agreement with previous finding by Speliotes et al [19], and with the hypothesis that the GCKR rs780094 SNP could lead to a higher activity of liver glcokinase. In addition we also observed higher triglycerides levels in Sicilian compared with Center/Northern Italy cohort, probably attributable to the more unhealthy lifestyle characterizing the southern population. By contrast we did not identify any association between severity of steatosis and GCKR genotype. Our data are not in contrast with the study of Speliotes et al [19] and more recent studies showing a link between GCKR SNP and the presence of steatosis [20]–[22]. We did not test subjects without steatosis of the same geographic area, and therefore we were not able to discriminate between presence/absence of fatty liver infiltration. Obviously, the lack of these data could partially affect the interpretation of our results, and in particular of the significance of the real effect of GCKR SNP on steatosis.

Finally, we did not confirm the reported association between PNPLA3 genotype and severity of liver fibrosis [14]. Differences in the characteristics of the individual study cohorts could explain the lack of association in our cohort.

The main limitation of this study lies in its cross-sectional nature, making it impossible to dissect the temporal relation between genetic background and progression of liver disease over time. This issue should be tested in longitudinal analyses. A further methodological question is the potentially limited external validity of the results for different populations and settings. Our study included cohorts of Italian patients enrolled at tertiary care centers, who may be different, in terms of both metabolic features and severity of liver disease, from the majority of prevalent cases of NAFLD in the general population. However, validation of the results in two independent, largely different populations from South and Northern/Center Italy supports a general involvement of GCKR rs780094 C>T polymorphisms in NAFLD progression.

In conclusion, this study on a large cohort of patients with histological diagnosis of NAFLD, showed an independent link between GCKR SNPs and significant hepatic fibrosis. Further studies are needed to explore the pathogenic mechanisms underlying this association.
