# Correction: Glucokinase Regulatory Protein Gene Polymorphism Affects Liver Fibrosis in Non-Alcoholic Fatty Liver Disease

**DOI:** 10.1371/journal.pone.0092497

**Published:** 2014-03-10

**Authors:** The PLOS ONE Staff

An affiliation for the sixth author is incorrect, and an affiliation for the sixth author is missing. Stefania Boccia is affiliated with the following institutions: Section of Hygiene, Institute of Public Health, Università Cattolica del Sacro Cuore, Rome, Italy; IRCCS San Raffaele Pisana, Rome, Italy.
